# Two Cases of Phthisis Pulmonalis Successfully Treated with Digitalis

**Published:** 1802-07

**Authors:** William Heron Rogers

**Affiliations:** Gravesend


### *Two Cases of Phthisis Pulmonalis successfully treated with Digitalis; communicated by* Mr. William Heron Rogers, *of Gravesend.*

M. W. a woman, aged twenty-fix years, of a delicate habit, and at all times fubject to a cough, had been declining in her health for feveral months, and at laft fhewed a ftrong tendency to Phthisis Pulmonalis. There was a confiderable difcharge of vifcid phlegm from the lungs, and the cough at night precluded reft. No hæmorrhage fucceeded the frequent returns of cough and fpitting; but the pulfe became rapid, the head ached, the teeth acquired a peculiar whitenefs, and the face flufhed as it commonly does in hectic fever. A pain in the fide was very troublefome for many weeks, which at laft abated from the application of two or three blifters. Perfpirations came on in the morning, and the ftate of the patient increafed hourly in danger. A great many nitrous and faline medicines were given, opiates and demulcents, but all to very little effect. I had before been a witnefs of the efficacy of Digitalis, and was determined to make trial of it in this cafe. Accordingly, I began with giving twelve drops of the tincture* three times a day, gradually augmenting the dofe till the pulfe, or head, became fenfibly affected. The bowels were kept open by cooling laxatives, and at night a febrifuge draught given, with twenty drops of landanum, in order to allay irritation and procure reft. Three days after my patient was put upon this plan, I had the fatisfaction to obferve a change for the better.

December 30, 1800. The dofe of the tincture being increafed to thirty drops three times a day, the perfpiration became lefs, the pulfe got down to 60, and the cough was neither fo frequent nor fo violent. During this amendment the appetite did not return, nor could the bowels be kept open without the aid of folutive medicines. The Digitalis, however, always abated fever and allayed irritation.

January 4, 1801. Pulfe funk to 35. Cough not fo troublefome. Expectoration lefs. Breathing much eafier. Nights good.

The 7th. Pulfe the fame. Perfpiration gone off. Slept well. The tickling in the trachea, which had long excited uneafy refpiration,

* It was according to Dr. Drake's formula in the Medical and Phyfical Journal for *December*, 1800, page 523.

piration, and frequent ſpitting, ſubſided; and the caſe went on in all other reſpects as well as poſſible.

The 12th. Continued taking thirty drops of the tincture. Slept very well, and began to have an inclination for food, which conſiſted of milk and bread, Glouceſter jelly,* vegetables, beef tea, and other aliment of the lighteſt kind.

The 20th. Tincture continued. Pulſe ſtatu quo. Breathing free. Fever and perſpirations totally removed.

The 30th. Very cool. Skin ſoft and pleaſant. Appetite better. Good nights. As every diſagreeable ſymptom was now gone, and the ſtrength a good deal exhauſted, I ventured to give two ounces of plain decoction of bark three times a day, with twenty-five drops of the tincture of Digitalis in each doſe. The opiate was diſcontinued.

February 10th. Pulſe 50. Cough almoſt gone. Strength returning. Appetite good. Diminiſhed the doſe of the tincture to fifteen drops twice a day.

The 20th. Pulſe 60. Strength returning. Good nights. Doſe of the tincture ten drops night and morning.

The 30th. Pulſe natural. Cough gone. Appetite very good. Gentle exerciſe and a light nutritive diet were then enjoined for ſome time; and which, with the treatment above deſcribed, have had the happy effect of completely reſtoring the patient to good health.

——— A———s, twenty-ſeven years of age, of a conſumptive habit, and from infancy ſubject to cough and pain in the ſide, had been declining in health for three or four months, during which time the cough became worſe, and many ſymptoms of complete Phthiſis ſupervened. A thin figure, delicate complexion, high cheek bones and ſmall cheſt, conſtituted the natural form of this patient, and the occaſional fluſhing that now and then came on, pointed out a ſtrong inclination to true Phthisis.

She was the mother of two children; and during her pregnancy, enjoyed much better health than at any other period, but invariably had a return of cough and ſpitting after delivery. Going one day to a friend's houſe, ſhe caught a ſevere cold which brought on abortion, and had nearly ended in death. The loſs of blood from the premature birth of a ſix months child, reduced her to a very low ebb, and a hectic fever ſucceeding,

* ℞. Hord. perlat. ſago, orizæ, rad. eryng. aa ℥j. coque in aq. puræ lbvj. ad lbiij. colat. liquor. addend. lact. recent. lbj. ſacch. alb. q. s. capiatur ad libitum. This is ſo called from having had excellent effects in aſſiſting the recovery of the Duke of Glouceſter, after a long illneſs abroad, and which indeed is a moſt excellent nutritious jelly.

ceeding, threatened my patient with a ſpeedy diſſolution. In this ſtate of affairs, I was conſulted, and had little hopes of rendering any eſſential ſervice.

Pulſe on the 14th of December, 1801, 140. Spitting profuſe. Cough violent. Nights very bad. Frequent fluſhing. Colliquative perſpirations. Emaciated countenance. I inſtantly ordered twelve drops of the tincture of Digitalis to be given every ſix hours and an anodyne at bed-time, conſiſting of Aq. ammon. acet. ʒij. vin. antimon. tinct. opii aa gtt. xx. aq. puræ ℥jſs. ſyr. ʒj. and an aperient mixture to preſerve a free paſſage through the bowels.

On the 16th, the pulſe was lowered, but the cough and expectoration nearly the ſame. I ordered fifteen drops of the tincture for a doſe, and to be augmented two drops each time of taking till the head perceived the effect, or by the pulſe I ſhould deem it expedient to diminiſh the quantity. The 18th, pulſe 110. Cough not ſo frequent. Slept better. The 19th, pulſe 100. Cough rather more troubleſome. Expectoration diminiſhed. Sleep diſturbed. I increaſed the doſe of the anodyne. The 20th, pulſe 90. Cough better. Pain in the ſide almoſt gone. Slept well, took food, and ſat up one hour. The 22d, pulſe 58. Skin ſoft and warm. Perſpirations gentle. Nights good. Cough much better. Doſe of the tincture thirty drops once in ſix hours. The 24th, head rather painful. Dimneſs of ſight. At times ſick. Pulſe 40, extremely languid, and intermitting. I diminiſhed the doſe of the tincture to gtt. xxv. 6tis. horis; threw in more nouriſhment, and preſcribed a wine glaſsful of camphorated mixture to be taken when low. The 25th, pulſe 46. Nights good. Head eaſier. Cough removed. The 28th, pulſe ſtatu quo. Head perfectly eaſy. Spitting very trifling. Freedom of reſpiration. No thirſt nor colliquative ſweats. The 31ſt, pulſe nearly the ſame. Sat up the beſt part of the afternoon. Took ſome milk and jelly. Slept better, and was in good ſpirits. January 5th, 1782, pulſe 50. Appetite returning. Slept well. Spitting almoſt ſubſided. Coughed very ſeldom. The 12th, eat and ſlept well. Head eaſier. Pulſe 56. The 17th, pulſe ſtatu quo. Sickneſs gone off, and head eaſy. The 25th, very little cough. Acquired more ſtrength, and looked a great deal better. Pulſe 60. The 29th, pulſe 63. I now ordered twenty drops of the tincture with one ounce and a half of decoction of bark three times a day, and twenty drops of opium only at bed-time. February 4th, pulſe 66, and more regular. Diſcontinued the opiate, and took only twenty drops of the tincture night and morning. Sat up good part of the day and ate heartily. She improved daily in this plan; has not had the leaſt diſpoſition to a return of the the complaint; and on the 12th of February left off taking all medicines.

However much ſome Practitioners may decry the uſe of Digitalis, it certainly is one of the moſt valuable medicines in the Materia Medica; and if gentlemen were to give it fair trials in ſuch caſes as appear to be curable, I have no heſitation in ſaying that they would be of the ſame opinion with me, and acknowledge its efficacy in the cure of phthiſis, as well as in hæmorrhagy, dropſy, &c. I confeſs, that the tincture of this plant ſucceeded in theſe inſtances beyond my moſt ſanguine expectations; and with ſuch a remedy, I ſhall never deſpair of rendering great ſervice in caſes that are truly formidable, and of very long ſtanding.

Cicuta has its advocates in tuberculated phthiſis, and I have known it given with much benefit. I ſaw one caſe greatly relieved by Digitalis and Cicuta combined, which got worſe with other medicines. Bleeding at an early period is extremely ſerviceable, and now and then ſmall doſes of calomel when the pulſe and conſtitution will admit of it. The lancet, in my opinion, is frequently forgotten till adheſions have taken place, and hectic abſorption undermined the patient's ſtrength; an omiſſion which can never be ſurmounted by a ſeries of the moſt diligent attentions.

So liable as the lungs are to the impreſſions of atmoſpheric air, the Practitioner will have but little hopes of alleviating pulmonary affections, unleſs he adverts with particular regard to the properties of that vital fluid. He is aware that the ſubſtance of the lungs is delicate, and in ſome conſtitutions incapable of reſiſting the preſſure of an elaſtic body. The force of a column of air as it ruſhes into the bronchial tubes in inſpiration is conſiderable, and if the lungs are ab origine weak, ſmall arteries will ſooner or later burſt, and in all probability produce Phthiſis. In a prediſpoſed habit, ſome detriment always ſucceeds the continued operation of very cold or very unwholeſome air. In the one inſtance, re-action is greater than the ſtrength of the viſcus will allow, and in the other irritation gradually leads to obſtruction. Health cannot be preſerved without a general and extended motion of the lungs; nor can they reſiſt diſeaſe if the circulation through them be incomplete.

Conſtant dilatation, however, is very unfavourable to the removal of active complaints, and to this cauſe probably we are to aſſign the difficulty of treating pulmonary affections. Highly organized parts are ſoon thrown into exceſſive inflammation; and ſo much expoſed as the lungs are to the action of bodies ab externo, it is not any way ſurprizing that we ſee them frequently quently difeafed. It would be a happy circumftance if collapfe occurred oftener than it does; as a natural cure by adhefion in cafes of Hæmorrhagy would then take place; but, as a cough is the natural confequence of impeded breathing, it is extremely difficult to diminifh activity, and excepting where large evacuations are made, almoft impoffible to maintain paffive order. If this union be extenfive, the air and blood muft be greatly interrupted in their courfe through the lungs, and occafion the patient a great deal of uneafinefs The refpiration of thofe perfons who have received injuries in the cheft from the thruft of a fword, or mufket ball, is partially obftructed, and a large portion of the lungs thereby rendered ufelefs. Real Phthisis, I believe, like all other fcrophulous complaints, is of flow increafe, and perhaps firft called into action by external caufes inflaming the furface of minute veffels. Can it therefore be good practice to attempt a cure by ftimuli? The great enemy we know is inflammation, which can never be fubdued but by evacuations, quietude, and an antiphlogiftic plan.

*June* 9, 1802.

